# ALBSNN: ultra-low latency adaptive local binary spiking neural network with accuracy loss estimator

**DOI:** 10.3389/fnins.2023.1225871

**Published:** 2023-09-13

**Authors:** Yijian Pei, Changqing Xu, Zili Wu, Yi Liu, Yintang Yang

**Affiliations:** ^1^Guangzhou Institute of Technology, Xidian University, Xi'an, China; ^2^School of Microelectronics, Xidian University, Xi'an, China; ^3^School of Computer Science and Technology, Xidian University, Xi'an, China

**Keywords:** spiking neural networks, binary neural networks, neuromorphic computing, sparsity, visual recognition

## Abstract

Spiking neural network (SNN) is a brain-inspired model with more spatio-temporal information processing capacity and computational energy efficiency. However, with the increasing depth of SNNs, the memory problem caused by the weights of SNNs has gradually attracted attention. In this study, we propose an ultra-low latency adaptive local binary spiking neural network (ALBSNN) with accuracy loss estimators, which dynamically selects the network layers to be binarized to ensure a balance between quantization degree and classification accuracy by evaluating the error caused by the binarized weights during the network learning process. At the same time, to accelerate the training speed of the network, the global average pooling (GAP) layer is introduced to replace the fully connected layers by combining convolution and pooling. Finally, to further reduce the error caused by the binary weight, we propose binary weight optimization (BWO), which updates the overall weight by directly adjusting the binary weight. This method further reduces the loss of the network that reaches the training bottleneck. The combination of the above methods balances the network's quantization and recognition ability, enabling the network to maintain the recognition capability equivalent to the full precision network and reduce the storage space by more than 20%. So, SNNs can use a small number of time steps to obtain better recognition accuracy. In the extreme case of using only a one-time step, we still can achieve 93.39, 92.12, and 69.55% testing accuracy on three traditional static datasets, Fashion- MNIST, CIFAR-10, and CIFAR-100, respectively. At the same time, we evaluate our method on neuromorphic N-MNIST, CIFAR10-DVS, and IBM DVS128 Gesture datasets and achieve advanced accuracy in SNN with binary weights. Our network has greater advantages in terms of storage resources and training time.

## 1. Introduction

Courbariaux et al. (2015) proposed Binary Connect, which pioneered the study of binary neural networks. Binarization can not only minimize the model's storage usage and computational complexity but also reduce the storage resource consumption of model deployment and greatly accelerate the inference process of the neural network. In the field of convolution neural networks (CNNs), many algorithms have been proposed and satisfactory progress has been made. However, conventional quantization techniques end up in either lower speedup or lower accuracy because these works fail to dynamically capture the sensitivity variability in the input feature map values. Therefore, we are motivated to apply different levels of quantization for different feature map values. Some researchers have embarked on the study of mixed-precision algorithms, which has led to many hardware accelerator designs. Chang et al. (2021) designed a reconfigurable CNN processor, which can reconstruct the computing unit and the on-chip buffer according to the computing characteristics of the model with mixed-precision quantization. Jiang et al. (2020) designed the PRArch accelerator architecture which support both conventional dense convolution and aggregated sparse convolution and implement mixed-precision convolution on fix-precision systolic arrays. Song et al. (2020) proposed an architecture that utilizes a variablespeed mixed-precision convolution array. It can achieve a significant improvement in performance with a small loss of accuracy.

Spiking neural networks, as the third generation of neural networks, is a computational paradigm that simulates the biological brain based on the dynamic activation of binary neurons and event-driven (Illing et al., 2019; Tavanaei et al., 2019). Using the time sparsity of binary time series signals can improve the computational energy efficiency on special hardware (Mead, 1990; Xu et al., 2020). The combination of SNNs and binary networks has gradually attracted more and more attention (Srinivasan and Roy, 2019; Lu and Sengupta, 2020; Kheradpisheh et al., 2022). However, it is still a great challenge to train SNNs due to their non-differentiable activation function. In order to maintain good accuracy, some researchers choose to use pre-training to obtain parameters from artificial neural networks (ANNs) (Cao et al., 2015; Lu and Sengupta, 2020; Wang et al., 2020; Xu et al., 2022b). The pre-training of ANN gives up the advantage of SNNs in temporal and spatial information processing. In recent years, some studies have successfully trained binarized SNNs (BSNNs) directly. For example, Jang et al. (2021) used the Bayesian rule to train BSNNs directly, and Kheradpisheh et al. (2022) used time-to-first-spike coding in the direct training of the network.

To maintain the energy efficiency and reasonable recognition accuracy of BSNNs, we propose accuracy loss estimators (ALE) and binary weight optimization (BWO). We use them to construct an ultra-low latency adaptive local binary spiking neural network. In addition, we apply global average pooling (GAP) structures to improve the speed of the networks further. To illustrate the superiority of our model, we conduct experiments on several datasets, our model dramatically improves the performance of BSNNs, and our contributions can be summarized as follows:

- Inspired by the mixed weight training, we design the ALE. When the network is trained, ALE will automatically select binary weight or full precision weight for training to solve the problem of large precision loss in the full binary weight training.
- We use the GAP layer instead of the fully connected layer to reduce the amount of calculation and change the output layer of SNNs to alleviate the phenomenon that it takes a long time to train BSNNs directly.
- To reduce the error caused by the binary weight in the backpropagation, we propose the BWO, which can directly adjust the binary weight based on the error. This method further reduces the error of networks and improves their performance.

## 2. Related works

### 2.1. Binary spiking neural networks

Generally, when choosing the quantization of the network, we can consider the following two aspects: weight and input (Qin et al., 2020). However, due to the characteristics of SNNs, there is no need to apply extra additional quantization of the network input. Recently, the idea of combining SNN and binarization has been proposed. Lu and Sengupta (2020) proposed B-SNN, which is transformed into BSNNs by pre-training binarized convolution neural network (BCNN). Roy et al. (2019) analyzed the results of combining different binary neurons with various binarized weight methods. Kheradpisheh et al. (2022) proposed BS4NN and explored the adaptation of simple non-leaky integrate-and-fire neurons, time-to-first-spike coding, and binarized weight in backpropagation. Jang et al. (2021) proposed BISNN, which combined Bayesian learning to train SNNs with binarized weights.

Guo et al. (2022) proposed a hardware-friendly local training algorithm. Binary random weights in the local classifiers were demonstrated to be effective in training without accuracy loss, which simplifies the algorithm for low-cost hardware implementation.

However, a lot of studies have focused on approximating full precision weights or reducing gradient errors to learn discrete parameters. For BSNN, it is usually to keep the first and last layers not binarized to reduce the accuracy drop based on the experimental experience (Deng et al., 2021). This method usually works, but there is still room for improvement.

### 2.2. Training of binary spiking neural networks

The training methods of BSNNs are also getting more and more attention. Recently, Mirsadeghi et al. (2021) proposed the STiDi-BP algorithm to avoid reverse recursive gradient computation while using binarized weights to obtain good performance. Wang et al. (2020) proposed the weights-thresholds balance conversion method to scale the full precision weights into binarized weights through changing the corresponding thresholds of spiking neurons and then effectively obtain BSNNs. Roy et al. (2019) trained ANNs with constrained weights and activations and deployed them into SNNs with binarized weights. The BS4NN proposed by Kheradpisheh et al. (2022) takes the advantage of the temporal dimension and performs better than a simple binary neural network with the same architecture.

Che et al. (2022) developed a differentiable hierarchical search framework for spiking neurons, where spike-based computation is realized on both the cell and the layer level search space. Guo et al. (2023) has studied what roles the temporal truncation and local training play in affecting accuracy and computational cost including GPU memory cost and arithmetic operations. Zhao et al. (2022) proposed a more biologically plausible spike timing dependent plasticity routing mechanism. Yang et al. (2022) proposed a novel spike-based framework with minimum error entropy and used the entropy theory to establish the gradient-based online meta-learning scheme in a recurrent SNN architecture.

The current BSNNs training method mainly uses all binarized weights, which fails to achieve a balance between accuracy and spatial quantization. Furthermore, SNNs usually require sufficient time steps to simulate neural dynamics and encode information and also take a long time to converge, which brings huge computational costs (Sengupta et al., 2019).

## 3. Methods

In this section, we will first introduce the neuron model, binary spiking neural network learning method, and GAP Layer and binarization method. Then, we will also introduce our proposed accuracy loss estimator and binary weight optimization.

### 3.1. Iterative leaky integrate-and-fire neural model

In this study, we use the iterative leaky integrate-and-fire (LIF) neuron model to construct networks. First, we will introduce the classic leaky integrate-and-fire model, which is defined as


(1) $$\begin{array}{l} \tau \frac{du\left(t\right)}{dt}=-u\left(t\right)+I\left(t\right),u<V_{th}, \end{array}$$

where *u*(*t*) is the membrane voltage of the neuron at time *t*, τ is the decay constant of the membrane potential, and *I*(*t*) is the input from the presynaptic neuron. The membrane potential *u* exceeds the threshold *V*_*th*_ and then returns to the resting potential after firing a spike. Then, the LIF neuron model is converted into an iterative version that is easy to program. Specifically, an iterative version can be obtained by the last spiking moment and the presynaptic input:


(2) $$\begin{array}{l} u\left(t_{i}\right)=u\left(t_{i-1}\right)e^{\frac{t_{i-1}-t}{\tau }}+I\left(t_{i}\right), \end{array}$$

where *u*(*t*_*i*−1_) is the membrane voltage at time step *t*_*i*−1_ and the *I*(*t*_*i*_) is the input from the presynaptic neuron at time step *t*_*i*_.

When the neuron output is zero before the last moment, the membrane voltage leaks. This process can be expressed mathematically simply:


(3) $$\begin{array}{l} u_{p}^{l+1}\left(t_{i+1}\right)=\tau u_{p}^{l+1}\left(t_{i}\right)\left(1-o_{p}^{l+1}\left(t_{i}\right)\right)+\overset{l_{max}}{\underset{q=1}{\sum }}w_{pq}o_{q}^{l}\left(t_{i+1}\right), \end{array}$$

where $u_{p}^{l+1}\left(t_{i+1}\right)$ is the membrane voltage of *p*th neuron of (*l*+1)th layer at time step *t*_*i*+1_, $o_{p}^{l+1}\left(t_{i}\right)$ is the output of *p*th neuron of (*l*+1)th layer at time step *t*_*i*_, τ is the decay factor, *w*_*pq*_ represents the weight of the *q*th synapse to the *p*th neuron, and *l*_*max*_ is the total number of neurons at the *l*th layer.

Finally, a step function *f*(*x*) is used to represent whether the neuron's membrane voltage reaches a threshold voltage *V*_*th*_ and fires a spike:


(4) $$\begin{array}{l} o_{p}^{l+1}\left(t_{i+1}\right)=f\left(u_{p}^{l+1}\left(t_{i+1}\right)\right), \end{array}$$

where the step function is $f(x)=\{\begin{array}{cc} 1 & x\geq \;V_{th}\\ 0 & x<V_{th} \end{array}$

### 3.2. Accuracy loss estimator for weight binarization

To reduce the accuracy drop of BSNNs, it is usually to keep the first and last layers non-binarized based on engineering experience, which means that the weight precision of the first and last layers plays an important role in the inference of the neural network (Deng et al., 2021). However, according to our study, which layer should be binarized depends on the structure of the neural networks and the characteristics of the datasets, and it is not always the best solution to keep the first and last layers with full precision.

As shown in Table 1, under the same binary network structure of Fashion-MNIST and CIFAR-10, scheme 1: keep the first and last layers with full precision, and scheme 2: keep the weights of the first two layers of the network as full precision. The result of scheme 2 is better than that of scheme 1.

**Table 1:** Accuracies from different methods.

**Dataset**	**Network architecture**	**High precision layer**	**Acc(%)**
Fashion-MNIST	Structure-1	Scheme 1	92.42
Fashion-MNIST	structure-1	Scheme 2	93.01
CIFAR-10	Structure-2	Scheme 1	85.91
CIFAR-10	Structure-2	Scheme 2	86.43

Therefore, we propose ALE, which automatically selects binarized and non-binarized network layers during network training by estimating the effect of different network layers on network accuracy.

First of all, we used the Manhattan distance between approximate binarized weights and full precision weights as the error estimation of binarized weight $w_{loss}^{l}$, and its calculation formula is shown below:


(5) $$\begin{array}{l} w_{loss}^{l}=\overset{n}{\underset{i=1}{\sum }}|w_{i}^{l}-bw_{i}^{l}|,l=1,2,3...L, \end{array}$$

where $w_{i}^{l}$ is the *i*th full precision weight of the *l*th layer and $bw_{i}^{l}$ is the *i*th approximate weight of the *l*th layer.

For a BSNN, each output channel of the spiking convolution layer corresponds to one feature extraction. So, we used the average error of feature extraction *A*^*l*^ to estimate the error caused by the binarized weights. The formula is shown below.


(6) $$\begin{array}{l} A^{l}=\frac{w_{loss}^{l}}{c_{out}^{l}}, \end{array}$$

where $c_{out}^{l}$ is the number of output channels of the *l*th layer.

There is a situation that is worth noting. If the error values *A*^*l*^ of the two layers in the network are similar and there is a significant difference in the number of weights, we certainly want to choose the one with more weights for binarization because it will save more space. Therefore, in addition to the error caused by binarization, we also consider the size of weight storage space as the criteria for selecting binarized layers, and the layer with a more significant number of weights will have a greater probability of being chosen for binarization.

Because error estimation *A*^*l*^ caused by binarization is calculated based on *w*_*loss*_ and *c*_*out*_, we tried to use them to estimate the difference in the weight storage space of different layers, the formula is as follows:


(7) $$\begin{array}{l} M^{l}=\frac{\theta_{max}^{l}-\theta_{1}^{l}}{2} \end{array}$$

$\theta_{max}^{l}$ is the *A*^*l*^ obtained when the number of output channels of the *l*th layer is equal to 1 and $\theta_{1}^{l}$ is the obtained *A*^*l*^ when the number of output channels of the *l*th layer equal to the total number of weights. For example, for a weight in the shape of [*output channel, input channel, kernel size, kernel size*] = [10, 10, 3, 3] its θ_*max*_ is equal to *A*^*l*^ in the shape of [1, 100, 3, 3], and θ_1_ is equal to *A*^*l*^ in the shape of [100, 1, 3, 3]. These *A*^*l*^ can be obtained quickly by using the Equations (5), (6).

To simplify the calculation of *M*, we used the *A*^*l*^ to estimate $\theta_{1}^{l}$ and $\theta_{max}^{l}$ based on the relationship between the error estimation of binarization weights with different shapes, which is obtained by experiments. The relationship is shown below.


(8) $$\begin{array}{l} \frac{w_{loss}^{l}}{w_{loss}^{l^{\prime }}}\approx \sqrt[{2}]{{\left(\frac{c_{out}^{l}}{c_{out}^{l^{\prime }}}\right)}^{2}*\frac{c_{in}^{l}}{c_{in}^{l^{\prime }}}}, \end{array}$$

where $w_{loss}^{l}$, $c_{out}^{l}$, and $c_{in}^{l}$ are the weight error of *l*th layer, the number of output channels, and the number of input channels, respectively. $w_{loss}^{l^{\prime }}$, $c_{out}^{l^{\prime }}$, and $c_{in}^{l^{\prime }}$ are the weight error of *l*th layers reshaped weights, the corresponding number of output channels, and the corresponding number of input channels, respectively.

**Figure 1:**
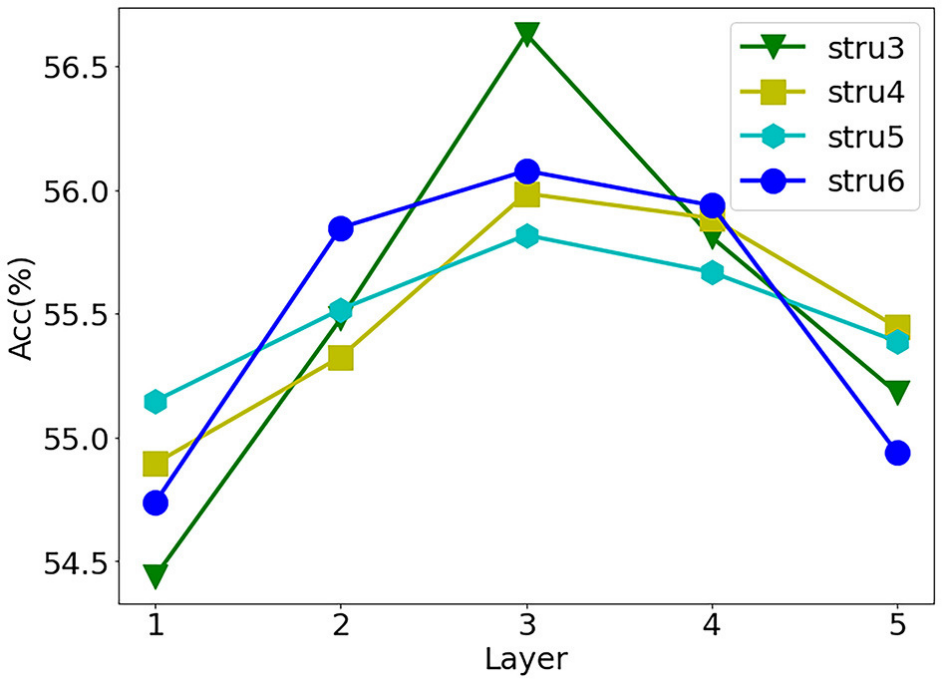
Influence of different binary layers on accuracy. On Cifar10, based on structure-3, we translate the precision curves under other structures (structure-4,5,6). Abscissa is the subscript of the binarization layer (the weights of other layers keep high precision), the first subscript is 1, and the ordinate is result accuracy.

Furthermore, we consider the influence of binarized weights at different layers in the forward pass and backpropagation. We set the same number of weights in each layer and carried out binarization layer by layer, and the network structure (structure-3,4,5,6) is shown in Table 2. At the same time, we observe the impact of the binary weights of each layer on the network recognition accuracy. Due to the first and second layers having been proven to have a significant influence on the accuracy of networks (Qin et al., 2020), we only study the weights of other layers. As shown in Figure 1, the network accuracy decreases even more when the layers at both ends of the network use binary weights.

**Table 2:** Network structure of different methods.

**Name**	**Network architecture**
Structure-1	16C3-16C3-AP2-64C3-64C3-AP2-256C3-1024C3-10
Structure-2	16C3-32C3-AP2-512C3-AP2-512C3-1024C3-10
Structure-3	10C3-10C3-10C3-10C3-10C3-10C3-10
Structure-4	16C3-16C3-16C3-16C3-16C3-16C3-10
Structure-5	30C3-30C3-30C3-30C3-30C3-30C3-10
Structure-6	50C3-50C3-50C3-50C3-50C3-50C3-10

We can take the subscript of the middle layer as the central axis, set the importance of the first and last layers to η, and use an approximate parabola to describe this phenomenon:


(9) $$\begin{array}{l} F\left(x\right)=\epsilon {\left(x-\frac{sumL+1}{2}\right)}^{2}, \end{array}$$

where *x* is the index of layer, ϵ is a facter which is equal to $\frac{4*\eta }{{\left(sumL-1\right)}^{2}}$, *sumL* represents the total number of layers, η is a variable, and we set it to 1 by default.

We combine *A*^*l*^, *M*^*l*^, and *F*(*x*) together to get the criteria *R*(*x*) for selecting binarized layers, which is shown below.


(10) $$\begin{array}{l} \begin{array}{l} R(x)=\left\{ \begin{array}{l} \left(\frac{1}{A^{l}+M^{l}}\right)F(x)\;,x\leq \frac{sumL+1}{2}\\ \left(\frac{1}{A^{l}+M^{l}}\right)log_{10}(K)F(x)\;,\;x>\frac{sumL+1}{2}, \end{array}\right. \end{array} \end{array}$$

where *K* represents the number of classes in the dataset. We can make different selection strategies according to the value of *R*(*x*) to satisfy different applications. We will discuss the strategies in detail in the experiment section.

### 3.3. GAP layer

Because of the binary output of spiking neurons, it is extremely sensitive to noise when the results of a few time steps are directly used for classification. Therefore, it is usually to use the spiking trains for a long period of time to indicate the degree of response to the category, which causes extra computational consumption. To address this problem, we learn from CNN's global average pooling (Lin et al., 2013) and apply it in SNNs to reduce the time steps.

**Figure 2:**
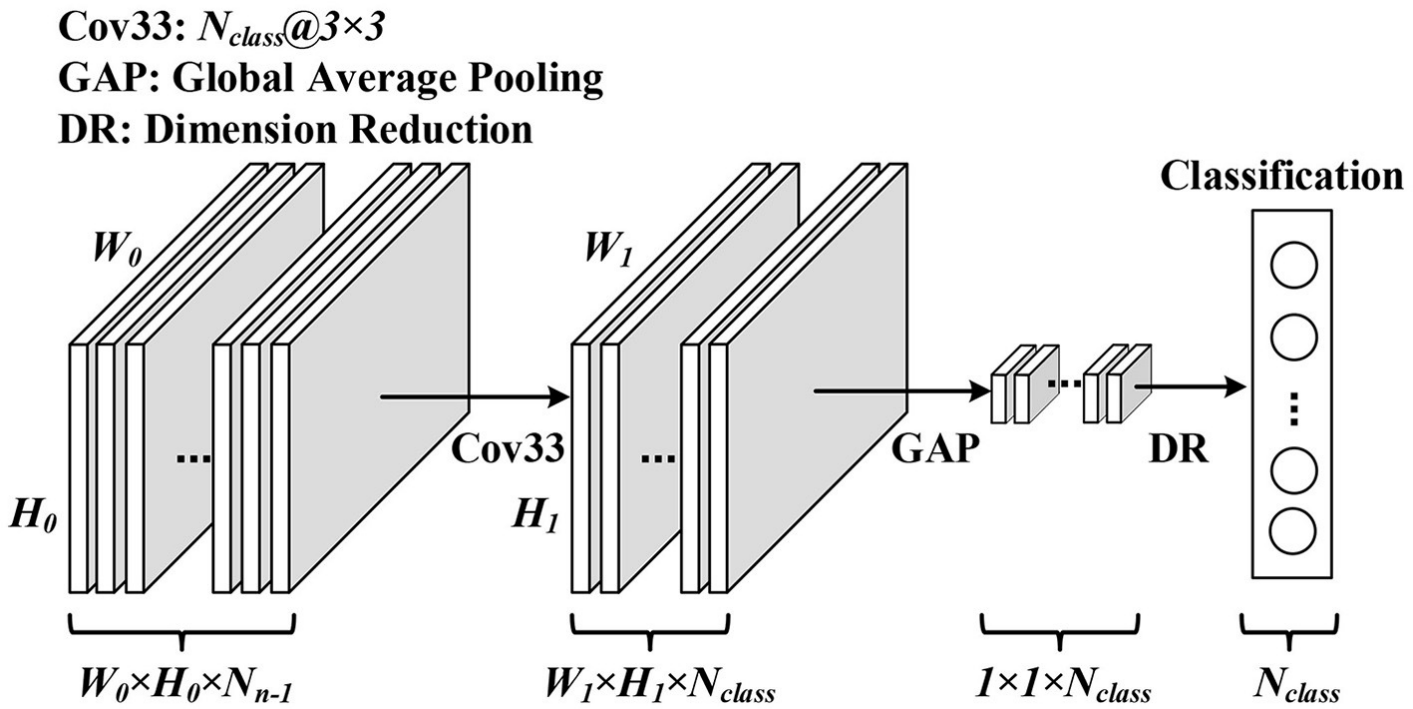
Overall structure of the GAP layer.

The GAP layer consists of a convolutional layer and a global average pooling layer (GAP) (Lin et al., 2013). The convolution layer adjusts output channels to the number of classifications of the dataset. The global average pooling layer converts the feature map into a classification vector, which is directly related to the final classification result. The overall structure of the GAP layer is shown in Figure 2. The number of output channels is first adjusted to the number of dataset classes by convolution calculation. Then, a global average pooling is used to transform the spatial average of the feature maps from the last layer to the confidence of categories. The obtained confidence is used as the probability of recognition. Just as GAP plays a role in CNNs, it can enforce correspondence between feature maps and categories and integrates global spatial information of SNNs.

### 3.4. Backpropagation with adaptive local binarization

For the binarization of the weights, we use three binarized weight blocks for the binarization approximation of the full precision weights. That is, a linear combination of three binary filters α is used to represent the full precision weight *W*.


(11) $$\begin{array}{l} W\approx \alpha_{1}B_{1}+\alpha_{2}B_{2}+\alpha_{3}B_{3}. \end{array}$$

In this way, ALE's formula 5 for calculating *w*_*loss*_, in which *bw* is transformed into $bw=\overset{3}{\underset{i=1}{\sum }}|\alpha_{i}W_{i}|$.

Then, we calculate the value of each binarized weight *B* referring to Lin et al. (2017). The equations are given as follows:


(12) $$\begin{array}{l} B_{i}=sign\left(W-mean\left(W\right)+\left(i-2\right)std\left(W\right)\right),i=1,2,3, \end{array}$$

where *mean*(*W*) and *std*(*W*) are the mean and standard deviation of *W*, respectively.

Once *B* is obtained, we can get α easily according to


(13) $$\begin{array}{l} \underset{\alpha }{min}J\left(\alpha \right)=||w-B\alpha |{|}^{2} \end{array}$$

For the forward pass, the forward calculation rule of approximate convolution in Lin et al. (2017) is still used, but the network needs to choose whether to binarize the weight of which layer according to ALE, instead of artificially fixing the binarization layer. The forward propagation formula is as follows:


(14) $$\begin{array}{l} O=\left\{ \begin{array}{l} \sum_{m=1}^{3}\alpha_{m}Conv(B_{m},A)\;Binarization\\ Conv(W,A)\;else \end{array}\right. \end{array}$$

where *Conv*() represents convolution function and *A* and *O* are the input and output tensor of a convolution, respectively.

BSNNs are affected by binarized weight and binary input, so the backpropagation process must be reconsidered. We use the Dirac function to generate the spikes of SNNs. Due to the non-differentiability of the Dirac function, the approximate gradient function is used instead of the derivative function in backpropagation (Wu et al., 2018; Neftci et al., 2019; Xu et al., 2022a), the approximate gradient function is defined as follows:


(15) $$\begin{array}{l} h\left(u\right)=\frac{1}{a}sign\left(\left|u-V_{th}<\frac{a}{2}\right|\right), \end{array}$$

where *u* represents the membrane voltage, *V*_*th*_ represents the threshold, and *a* is the parameter that determines the sharpness of the curve.

Using the chain rule, the error gradient with respect to the presynaptic weight W is


(16) $$\begin{array}{l} \frac{\partial L}{\partial W}=\frac{\partial L}{\partial O}\frac{\partial O}{\partial W}=\frac{\partial L}{\partial O}\left(\frac{1}{a}sign\left(\left|u-V_{th}<\frac{a}{2}\right|\right)\right), \end{array}$$

where *L* is the loss function and *sign* is signum function.

Moreover, the binarization function of weight is also a typical step function, and a straight-through estimator (STE) (Bengio et al., 2013) is usually used to solve this problem.


(17) $$\begin{array}{l} \frac{\partial L}{\partial W}\overset{STE}{=}\frac{\partial L}{\partial O}\frac{\partial O}{\partial B}\frac{\partial Htanh}{\partial W}=\frac{\partial L}{\partial O}\frac{\partial O}{\partial B}=\frac{\partial L}{\partial B} \end{array}$$

where *O* and *Htanh* as the output tensor of a convolution and hard-tanh function, respectively.

**Figure 3:**
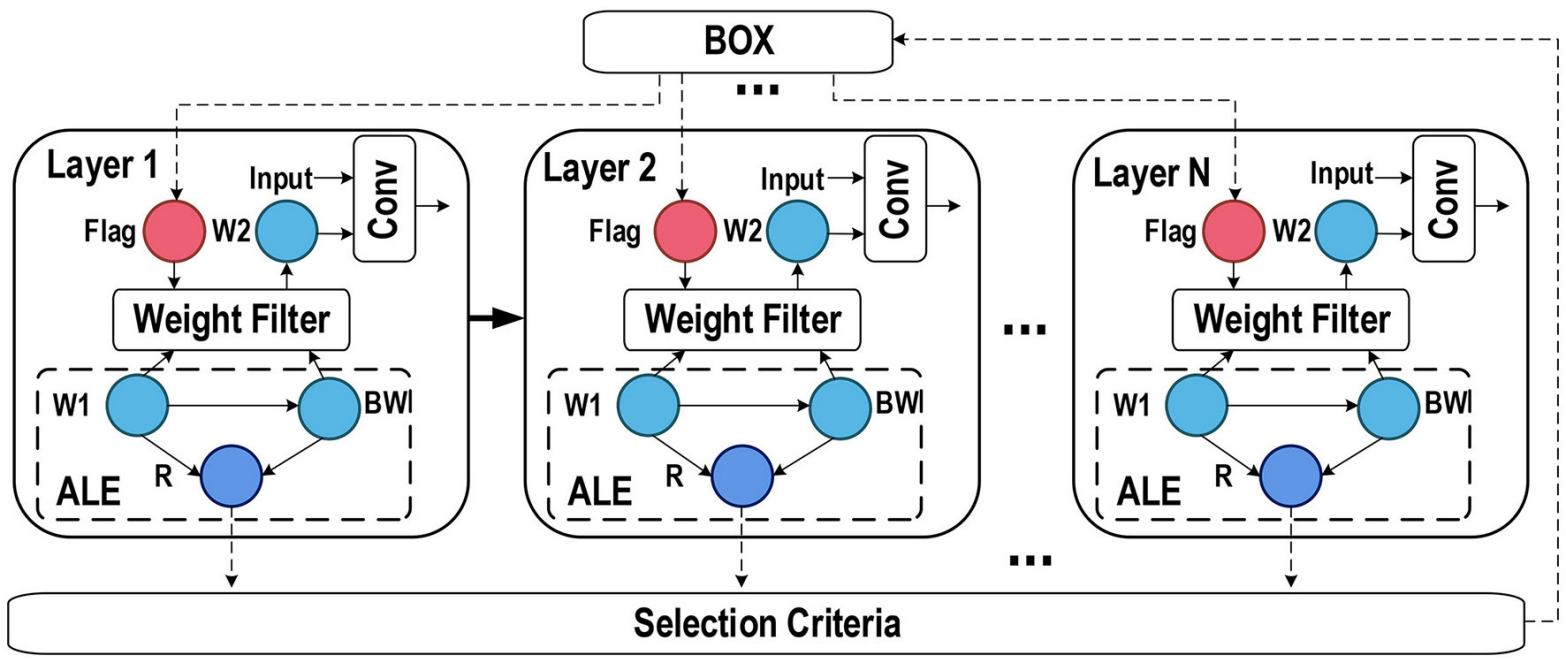
Network layers with ALE. The box records the index of layers that need to be binarized. The flag determines whether the binarized weights are used for convolution calculation. *W*1, *BW*, and *W*2 represent the original weight, the binarized weight, and the weights selected for convolution calculation, respectively. *Conv* is the convolution function.

In Figure 3, we show the network layer with ALE and its workflow. First, the network can use the *Flag* obtained from “Box” to determine whether this layer uses binarized weights. Then, the selected weights are convolved with the input. For the current training step, “Box” stores the selection result of the last training step, and these results will be used to select whether the binarized weight will be used. ALE will recalculate the value of *R* and update the selection results in the “Box” simultaneously. Next, the process for ALE to recalculate the value of *R* is as follows. It calculates the binarized weight *BW* according to the original weight *W*1, and then they work together to get *R*. Finally, the selection result depends on the value of *R* and the selection criteria, and the results are updated to the ‘Box'.

**Figure 4:**
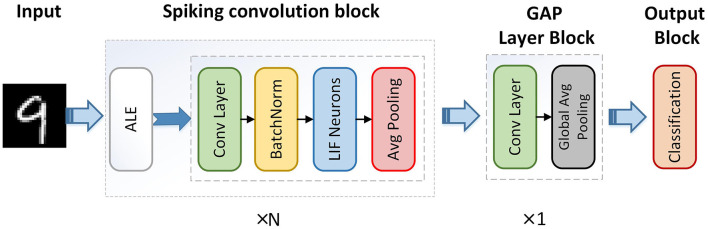
Overall structure of adaptive local binary spiking neural network.

Therefore, the overall structure of the adaptive local binary Spiking Neural Network (ALBSNN) structure is illustrated in Figure 4. The network consists of *N* end-to-end spiking convolution blocks and a GAP layer block. The spiking convolution block consists of an ALE, a spiking convolution layer, a batch normalization layer, and an average pooling layer. ALE decides whether the weight is binarized or not, and the spiking convolution layer extracts the features of the image. The GAP layer is used to alleviate the excessive cost of the time steps.

### 3.5. Binary weight optimization

We use three binarized weight blocks for the binarization approximation of full precision weights, and it is classified as the problem of solving the optimal weight coefficient. When the neural network training tends to be stable, the binary weight processed by the sign function is almost difficult to change. For the network that reaches the training bottleneck, coefficient optimization can no longer meet the demand for improving accuracy. However, the accuracy can be further improved by adjusting the binary weight.

**Figure 5:**
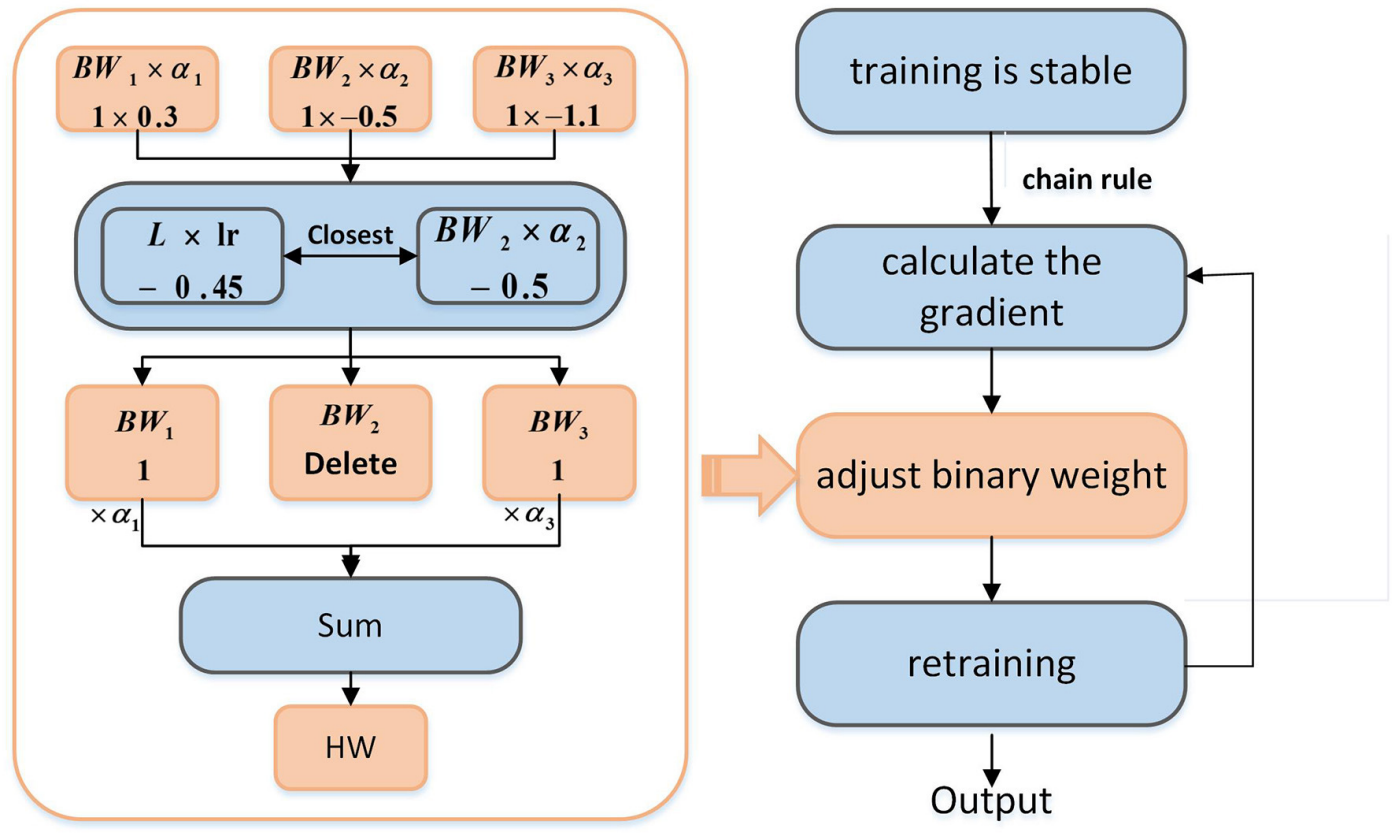
Overall structure of binary weight optimization.

To keep the degree of adjustment controllable, we modify only one binary weight to meet the demand for weight change. As shown in Figure 5, when the network training is stable, *L* is the gradient calculated according to the chain rule, and its product with the learning rate *lr* is the adjustment on a single weight. Because the weight is composed of three binary weights, we choose one of the binary weights, which needs to meet the condition that among these binary weights *BW*_*i*_(*i* = 1, 2, 3), *BW*_*i*_×α_*i*_ is the closest to the adjustment (*L*×*lr*). Then, delete this binary weight *BW* and its coefficient α, that is, the weight is only composed of the remaining two binary weights.

Two more restrictions are required for the above methods: (1) There is a situation in which we do not update the binary weight. If *BW*×α is much larger than *L*×*lr*, the update of the binary weight will cause more errors resulting in accuracy degradation of the network. Therefore, the selected “closest” binary weights need a restriction to determine whether the weights are adjusted. In this article, we stipulate that the difference between *L*×*lr* and *BW*×α must not exceed *L* 100 times. Otherwise, the selected “closest” binary weight will not be adjusted. (2) Only adjust the network layer using binary weights.

Finally, as shown in Figure 5, the adjusted binary weights will be recombined into full precision weights, and it needs to be trained again to make the weight better adapted to the network. A profit can be obtained by doing a small amount of binary weight optimization.

## 4. Experiments

In this section, we evaluate our proposed adaptive local binary spiking neural network (ALBSNN) on both traditional static Fashion-MNIST (Xiao et al., 2017), CIFAR-10, and CIFAR-100 (Krizhevsky et al., 2009) datasets and neuromorphic N-MNIST (Orchard et al., 2015), CIFAR10-DVS (Li et al., 2017), and DVS128 Gesture datasets (Amir et al., 2017). Fashion-MNIST is a fashion product image dataset with 10 classes, 70,000 grayscale images in the size of 28 × 28. CIFAR-10 and CIFAR-100 are composed of three channel RGB images of size 32 × 32. CIFAR-10 has 10 classes, while CIFAR-100 has 100 classes, and all images are divided equally by class. The neuromorphic-MNIST (N-MNIST) dataset is a spiking version of the MNIST dataset recorded by the neuromorphic sensor. It consists of 60,000 training examples and 10,000 test examples. CIFAR10-DVS is composed of 10,000 examples in 10 classes, with 1,000 examples in each class. DVS128 Gesture dataset contains 11 kinds of hand gestures from 29 subjects under three kinds of illumination conditions.

### 4.1. Experimental setup

All reported experiments below are conducted on an NVIDIA Tesla V100 GPU. The implementation of our proposed ALBSNN is on the Pytorch framework (Paszke et al., 2019). Only one timestep is used to demonstrate the advantage of our proposed ALBSNN on ultra-low latency. Adam is applied as the optimizer (Kingma and Ba, 2014). The results shown in this study refer to the average results obtained by repeating five times.

In this study, we apply several data augmentation during training processing as follows: (1) padding the original figure, and the padding size is 4, (2) crop pictures with a size of 32 pixels randomly, (3) flip the image horizontally with half probability, and (4) normalized image, the standard deviation is 0.5. For the testing process, only normalization is applied (Shorten and Khoshgoftaar, 2019).

We use an iterative LIF model and approximate gradient for network training. The first convolutional layer acts as an encoding layer and network structures for Fashion-MNIST, CIFAR-10, CIFAR-100, N-MNIST, DVS128 Gesture, and CIFAR10-DVS datasets are shown in Table 3. Between the convolution calculation and the activation function, batch-normalization(BN) (Ioffe and Szegedy, 2015) is applied. All convolution operations used in the experiment are based on the operations provided by Pytorch. The hyperparameters of networks we used in our experiments are shown in Table 4. The learning rate uses the cosineanealing strategy (Loshchilov and Hutter, 2016). Unless otherwise specified, our experiments report the testing accuracy of Fashion-MNIST, N-MNIST, CIFAR-10, CIFAR10-DVS, and DVS128 Gesture after training 50 epochs. For CIFAR-100, 400 epochs are applied for training.

**Table 3:** Network structures.

**Dataset**	**Structure**
*MNIST	16C3-16C3-AP2-64C3 -64C3-AP2-256C3-1024C3-GAP
*CIFAR-10	128C3-256C3-AP2 -512C3-AP2-1024C3-512C3-GAP
CIFAR-100	128C3-256C3-AP2-512C3 -AP2-1024C3-512C3-512C3-GAP

*MNIST represents Fashion-MNIST and N-MNIST datasets. *CIFAR-10 represents CIFAR-10, DVS128 Gesture and CIFAR10-DVS datasets.

**Table 4:** Parameters setting.

**Parameter**	*MNIST	*CIFAR-10	**CIFAR-100**
*V*_*th*_	0.5	0.5	0.5
τ	0.25	0.25	0.25
a	1	1	1
Learning rate	0.001	0.001	0.001
Batch size	16	16	16
Time step	1	1	1
Optimizer	Adam	Adam	Adam
Criterion	MSE	MSE	Cross-Entropy

*MNIST represents Fashion-MNIST, and N-MNIST datasets. *CIFAR-10 represents CIFAR-10, DVS128 Gesture and CIFAR10-DVS datasets.

### 4.2. Effectiveness of ALE and BWO

To validate the effectiveness of ALE and BWO, we compare ALBSNN, SNN with full precision weights (FPSNN), SNN with binarization of all weights (BSNN), and BSNN whose first layer and last layer are non-binarized (FLNBSNN) on each dataset. For the fairness of comparison, ALBSNN is designed to select two layers to maintain full precision. Table 5 shows the accuracy of different methods. We obtain FPSNN and BSNN results by STBP (Wu et al., 2018) and ABC-NET (Lin et al., 2017). Compared with FPSNN, BSNN, FLNBSNN, and ALBSNN will drop some accuracy due to binarization. ALBSNN achieves better results in accuracy because the ALE block can help network select more suitable layers based on the network structure and dataset. In some datasets, the selection result of ALBSNN is the same as that of FLNBSNN, which is affected by the network structure. We will discuss it in the next section.

**Table 5:** Accuracy of different methods static datasets.

**Dataset**	**Method**	**Full precision layer**	**Acc(*%*)**
Fashion-MNIST	BSNN	-	92.38
	FLNBSNN	1,7	92.92
	ALBSNN	1,2	93.10
	ALBSNN + BWO	1,2	93.39
	FPSNN	all	93.48
CIFAR-10	BSNN	-	89.65
	FLNBSNN	1,6	91.01
	ALBSNN	1,6	91.64
	ALBSNN + BWO	1,6	92.12
	FPSNN	all	92.37
CIFAR-100	BSNN	-	59.98
	FLNBSNN	1,7	68.19
	ALBSNN	1,7	68.65
	ALBSNN + BWO	1,7	69.55
	FPSNN	all	70.00

To validate the effectiveness of binary weight optimization (BWO). Tables 5, 6 make a comparison of a binary network with and without BWO. We maintain the training environment of ALBSNN here without additional parameter adjustment. At the same time, we only use BWO to train the network 20 times on all datasets to avoid excessive consumption of network resources. On these datasets, binary weights are optimized further by the proposed BWO. The accuracy of the network on Fashion-MNIST, N-MNIST, DVS128 Gesture, and CIFAR-10 has almost reached the level of the full-precision network, so the improvement in accuracy is not particularly significant. For larger and more complex datasets, such as the CIFAR-100 and CIFAR10-DVS, our method has greater potential to improve accuracy.

**Table 6:** Accuracy of different methods on neuromorphic datasets.

**Dataset**	**Method**	**Full precision layer**	**Acc(%)**
N-MNIST	BSNN	-	98.38
	FLNBSNN	1,7	99.13
	ALBSNN	1,2	99.19
	ALBSNN + BWO	1,2	99.33
	FPSNN	all	99.40
DVS128 Gesture	BSNN	-	92.32
	FLNBSNN	1,6	94.55
	ALBSNN	1,6	94.77
	ALBSNN + BWO	1,6	95.33
	FPSNN	all	95.68
CIFAR10-DVS	BSNN	-	58.38
	FLNBSNN	1,6	68.01
	ALBSNN	1,6	68.31
	ALBSNN + BWO	1,6	68.98
	FPSNN	all	71.38

### 4.3. Rethink about local binarization

Compared with the selection results on each dataset, we find these selection results are related to the complexity of the dataset and the network structure. As shown in Tables 5, 6, ALBSNN chooses the same layers as FLNSNN to keep full precision when the structure used by the dataset is the *CIFAR-10 in Table 4. If we change the network structure so that the difference between the weights of the head layer and the tail layer is larger, then we will get different results from FLNBSNN. The network structure is shown in Table 7. ALBSNN chooses to keep the weight accuracy of the first and second layers to the full precision (weight binarization of other layers), and the network accuracy is higher than that of FLNBSNN.

**Table 7:** Different results of ALBSNN and FLNBSNN.

**Dataset**	**Network architecture**	**Method**	**Full precision layer**	**Acc(*%*)**
CIFAR-10	16C3-32C3-AP2-512C3-AP2 -512C3-1024C3-GAP	FLNBSNN	1,6	85.91
CIFAR-10		ALBSNN	1,2	86.43
DVS128 Gesture		FLNBSNN	1,6	89.15
DVS128 Gesture		ALBSNN	1,2	89.89

If the final output channel is relatively small and the size of weights between adjacent network layers is relatively large, ALBSNN may obtain a better binarization scheme by ALE. However, if the size of weights in the network increases or decreases gradually, FLNBSNN is a good solution. As the weights of common networks generally conform to the rule of flat change layer by layer, the selection of ALE tends to be similar to FLNB. Of course, if the non-binarized layers are not limited to two, ALE still can obtain a better binarization scheme by evaluating the error caused by the binarized weights. To sum up, the selection result of ALE is mainly related to the complexity of the dataset and the structure of the neural network.

### 4.4. Impact of selection criteria

In the previous section, in order to make a fair comparison with FLNBSNN, we select the two layers with the largest value *R* as full precision layers. In this section, we choose four different selection criteria SC1, SC2, SC3, and SC4 to show the impact of the selection criteria on the accuracy of ALBSNN. SC1 applies the mean value *R* of all layers as the baseline. When the value *R* of a layer is greater than the mean value, this layer is selected as the full precision layer. SC2 uses the *R* of the last layer as the baseline. If the *R* of a layer is greater than the baseline, and the layer is non-binarized. For SC3, the first and last layers are selected as full precision layers, and the mean of *R* of the other layers is set as the baseline; *R* of other layers exceeds the baseline, the layer is selected as the full precision layer. For SC4, the first and last layers are selected as full precision layers, and the layer closest to the average value of *R* excluding these two layers is also regarded as the full precision layer.

As Table 8 is shown, a different binarization scheme is obtained based on the network structure and dataset by ALE with the different selection criteria. It is obvious that the accuracy is positively correlated with the number of layers using full-precision weights. Among them, SC2 has a significant improvement in accuracy and takes up less resources, which is the most cost-effective. In practice, we can choose the appropriate selection criteria according to the requirements of accuracy and weight storage space.

**Table 8:** Accuracy of different selection criteria.

**Dataset**	**Selection criteria**	**Full precision layer**	**Acc(*%*)**
Fashion-MNIST	SC1	1	92.81
	SC2	1,6	93.10
	SC3	1,2,7	93.26
	SC4	1,3,7	93.21
CIFAR-10	SC1	1	90.36
	SC2	1,6	91.64
	SC3	1,2,6	91.71
	SC4	1,5,6	91.69
CIFAR-100	SC1	1	65.68
	SC2	1,6	68.65
	SC3	1,2,6	68.88
	SC4	1,5,6	68.89

### 4.5. Compared with other methods

In this section, we compare our ALBSNN with several previously reported state-of-the-art methods with the same or similar binarization SNN network. For a fair comparison, we replace the fully connected layer with the GAP Layer and build an ALBSNN based on a similar network structure for discussion. For the Fashion-MNIST, BS4NN (Kheradpisheh et al., 2022) is trained with a simple fully connected network, and Mirsadeghi et al. (2021) uses a higher-performance convolutional network for recognition (we denote this network by SSTiDi-BP). Both networks use temporal backpropagation for learning. For CIFAR-10 and CIFAR-100 datasets, the network structures used by Roy et al. (2019) and Wang et al. (2020) are both modified VGG network (Simonyan and Zisserman, 2014); we used Roy-SVGG10 and Wang-SVGG10 to denote these two networks, respectively. They do not train the SNN directly but instead use the method of ANN-to-SNN conversion.

For neuromorphic datasets, the SNN train with binary weights is relatively scarce, so we used high-precision SNN for comparison here. LISNN (Cheng et al., 2020) and TDNNA-BP (Lee et al., 2020) carried out experiments on N-MNIST. CSRN (He et al., 2020) carried out experiments on DVS128 Gesture. NeuNormSNN (Wu et al., 2019) and ASF-BP (Wu et al., 2021) carried out experiments on CIFAR10-DVS. Table 9 shows the corresponding experimental results.

**Table 9:** Comparison of different methods.

**Dataset**	**Method**	**Learning**	**Epoch**	**Timestep**	**Weight storage space** **(Normalized)**	**Acc(%)**
Fashion-MNIST	BS4NN	Spike-based BP	500	100	1.85	87.50
	SSTiDi-BP	Spike-based BP	-	100	3.09	92.00
	ALBSNN + BWO	Spike-based BP	20	1	1	92.04
CIFAR-10	Roy-SVGG10	ANN2SNN	150	-	1.26	88.27
	Wang-SVGG10	ANN2SNN	500	100	1.26	90.19
	ALBSNN + BWO	Spike-based BP	50	1	1	92.12
CIFAR-100	Roy-SVGG100	ANN2SNN	400	-	2.76	54.44
	Wang-SVGG100	ANN2SNN	500	300	1.18	62.02
	ALBSNN + BWO	Spike-based BP	400	1	1	69.55
N-MNIST	LISNN	Spike-based BP	20	100	5.86	99.45
	TDNNA-BP	Spike-based BP	100	50	2.92	99.09
	ALBSNN + BWO	Spike-based BP	50	10	1	99.27
DVS128 Gesture	CSRN	Spike-based BP	100	60	5.69	93.40
	ALBSNN + BWO	Spike-based BP	50	20	1	94.63
CIFAR10-DVS	NeuNormSNN	Spike-based BP	200	100	8.59	60.50
	ASF-BP	Spike-based BP	-	-	1.62	62.50
	ALBSNN + BWO	Spike-based BP	50	10	1	68.98

The weight storage space is normalized with respect to the baseline(ALBSNN). For traditional static datasets, our recognition accuracy is on the same level as state-of-the-art SNN networks with binary weights, but we use less training time and save more storage resources. Compared with Wang-SVGG10, our ALBSNN achieves 1.93 and 7.53% average testing accuracy improvement with only one-time steps and fewer epochs. For the weight storage space, our ALBSNN can obtain more than 20 and 15% reduction on the CIFAR-10 and CIFAR-100, respectively. For neuromorphic datasets, compared with the SNN network with high precision weights, our network still achieves advanced results, uses less training time, and saves more than 50% storage resources.

## 5. Conclusion

This study proposes a construction method of ultra-low latency adaptive local binary spiking neural network with an accuracy loss estimator, which balances the pros and cons between full precision weights and binarized weights by choosing binarized or non-binarized weights adaptively. Our network satisfies the requirement of network quantization while keeping high recognition accuracy. At the same time, we find the problem of long training time for BSNNs. Therefore, we propose the GAP Layer, in which a convolution layer is used to replace the fully connected layer, and a global average pooling layer is used to solve the binary output problem of SNN. Because of the binary output, SNN usually needs to run multiple time steps to get reasonable results. Finally, we find that when the BSNN is stable, the binary weight processed by the sign function is difficult to change, which leads to the bottleneck of network performance. Therefore, we propose binary weight optimization to reduce the loss by directly adjusting the binary weight, which makes the network performance close to the full-precision network. Experiments on traditional static and neuromorphic datasets show that our method saves more storage resources and training time and achieves competitive classification accuracy compared with existing state-of-the-art BSNNs.

## Data availability statement

Publicly available datasets were analyzed in this study. This data can be found here: data openly available in the public repository. The data that support the findings of this study are openly available in Fashion-MNIST at https://doi.org/10.48550/arXiv.1708.07747, CIFAR-10 at http://www.cs.utoronto.ca/~kriz/cifar.html, CIFAR-100 at http://www.cs.utoronto.ca/~kriz/cifar.html, CIFAR10-DVS at https://doi.org/10.3389/fnins.2017.00309, DVS128Gesture at https://research.ibm.com/interactive/dvsgesture/, and N-MNIST at https://doi.org/10.3389/fnins.2015.00437.

## Ethics statement

The studies were conducted in accordance with the local legislation and institutional requirements. Written informed consent for participation was not required from the participants or the participants' legal guardians/next of kin in accordance with the national legislation and institutional requirements because all the data in the study came from public datasets.

## Author contributions

YP, CX, and ZW contributed to conception and design of the study. YP and CX wrote the first draft of the manuscript. YY and YL use statistical, mathematical or other forms of techniques to analyze or synthesize research data. All authors contributed to manuscript revision, read, and approved the submitted version.
